# 5-Azacytidine partially restores CD20 expression in follicular lymphoma that lost CD20 expression after rituximab treatment: a case report

**DOI:** 10.1186/s13256-016-0809-7

**Published:** 2016-02-02

**Authors:** Yutaka Tsutsumi, Hiroyuki Ohigashi, Shinichi Ito, Souichi Shiratori, Takanori Teshima

**Affiliations:** 1 Department of Hematology, Hakodate Municipal Hospital, 1-10-1, Minato-cho, Hakodate, 041-8680 Japan; 2 Department of Hematology, Hokkaido University Graduate School of Medicine, N15 W7, Kita-ku, Sapporo, 060-8638 Japan

**Keywords:** 5-Azacytidine, CD20, Follicular lymphoma, Myelodysplastic syndrome

## Abstract

**Background:**

The loss of CD20 protein expression after a rituximab-containing regimen is one of the resistance mechanisms in non-Hodgkin's lymphoma. Recently, it was reported that 5-azacitidine administration upregulates the expression of CD20 in CD20-negative B-cell acute lymphoblastic leukemia. Here we report a similar upregulation in a patient with follicular lymphoma who was treated with 5-azacitidine against secondary myelodysplastic syndrome.

**Case presentation:**

A 69-year-old Japanese woman with follicular lymphoma with treatment-related myelodysplastic syndrome was negative for the CD20 antibody at the time of her relapse. After treatment of 5-azacytidine for her myelodysplastic syndrome, CD20 expression was upregulated in the follicular lymphoma cells in her peripheral blood. We also observed follicular lymphoma cell stimulation in her peripheral blood due to 5-azacytidine.

**Conclusions:**

Although partial, CD20 expression was upregulated after treatment with 5-azacitidine. However, CD20 expression was not re-upregulated after a second administration of 5-azacitidine and we also observed the risk of lymphoma cell stimulation due to 5-azacitidine.

## Background

Rituximab is a standard drug for the treatment of CD20-positive B-cell non-Hodgkin's lymphoma (NHL). The loss of CD20 protein expression after a rituximab-containing regimen is one of the resistance mechanisms in NHL [1, 2]. Recently, it was reported that 5-azacitidine (5-AZA) administration upregulates the expression of CD20 in CD20-negative B-cell acute lymphoblastic leukemia [3]. Here we report the case of a patient who was treated with 5-AZA against secondary myelodysplastic syndrome (MDS) that was caused by the treatment of B-cell follicular lymphoma (FL). CD20 expression was upregulated in CD20-negative FL cells after 5-AZA treatment.

## Case presentation

**Fig. 1:**
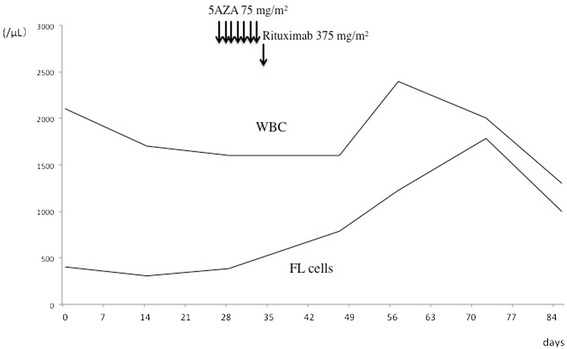
Clinical course of first 5-azacitidine treatment and changes of CD19 and CD10 double-positive lymphoma cells. *5AZA* 5-azacitidine, *FL* follicular lymphoma, *WBC* white blood cells

**Fig. 2:**
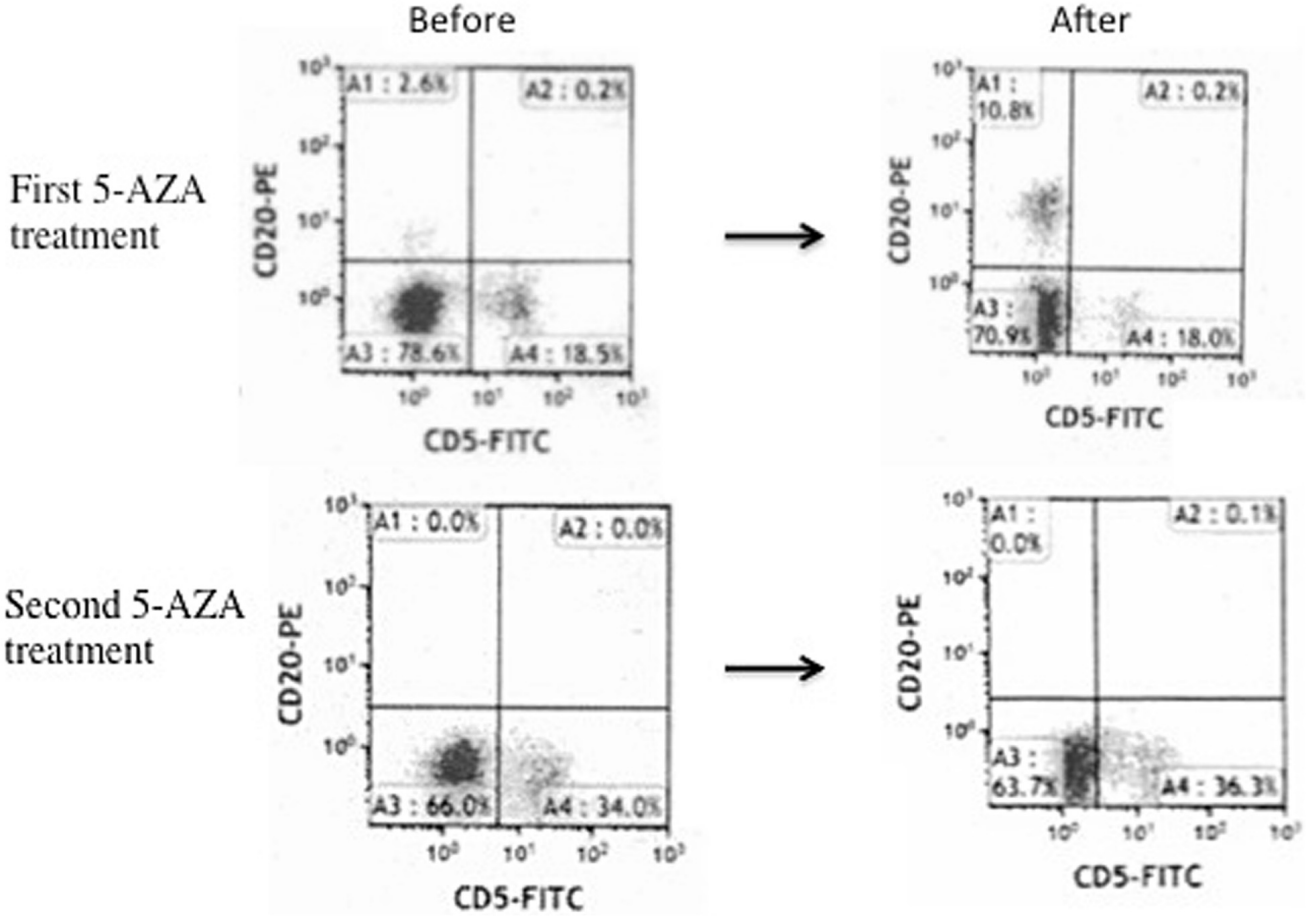
Seven-day administration of 5-azacitidine followed by 1-day rituximab treatment. CD20 expression restored in CD20-negative lymphoma cells but not in second course. *5AZA* 5-azacitidine

A 69-year-old Japanese woman developed grade 2 FL and exhibited symptoms that included swelling in her neck, pharyngeal tonsils, and axial and inguinal lymph nodes with FL invasion of her bone marrow. The lymphoma cells were also positive in her peripheral blood, and her clinical stage was IVA. Her FL international prognostic index (FLIPI) was assessed to be high risk based on her age, swollen lymph nodes, clinical stage IV, and lactate dehydrogenase (LDH) levels that were higher than the normal range. She had no pertinent family and psychosocial history. She was given six courses of R-THP-COP treatment (rituximab 375 mg/m^2^ on day 1, pirarubicin 30 mg/m^2^ on day 2, cyclophosphamide 500 mg/m^2^ on day 2, vincristine 1 mg/m^2^ on day 2, and prednisolone 30 mg/m^2^ on days 2 to 6) and achieved complete remission (CR). The disease recurred approximately 9 months later, and she was treated with a combination of bendamustine (90 mg/m^2^ for 2 days) and rituximab (375 mg/m^2^ for 1 day). She achieved partial remission (PR) and the FL cells disappeared: FL cells – CD10 and CD19 double-positive cells and IgH-BCL2 positive in polymerase chain reaction (PCR); however, in her peripheral blood (IgH-BCL2 negative by PCR and CD20-positive cell was negative in peripheral blood), FL recurred and a therapy-related MDS simultaneously developed 16 months after the diagnosis. Her pancytopenia and normal karyotype indicated a score of 2.5 points: blast cell 0.8 %; hemoglobin 11.1 g/dl; platelet count 6.7×10^9^/L; absolute neutrophil count 0.4×10^9^/L. Her revised international prognostic score showed an intermediate low risk. FL cell specimens were harvested from her peripheral blood. The CD20 protein expression in the lymphoid cells was analyzed by flow cytometry in which mouse anti-CD20 antibody (B2E9; Beckman Coulter, Fullerton, CA, USA) was used. The FL cells in her bone marrow and peripheral blood were CD20-negative. The clinical course of the first 5-AZA treatment is shown in Fig. 1. She was treated with 75 mg/m^2^ of 5-AZA against therapy-related MDS for 7 days. Of interest, 10.8 % of the CD20-negative lymphoma cells had restored CD20 expression after 5-AZA was administered (Fig. 2). Circulating FL cells also increased after its administration. We administered rituximab (375 mg/m^2^) 1 day after the last administration of 5-AZA to prevent the progress of FL. After administration of rituximab, we administered 375 mg/m^2^ of rituximab again and an 80 % dosage of a GDP regimen that contained 1000 mg/m^2^ of gemcitabine, 75 mg/m^2^ of cisplatin and dexamethasone 20 mg orally on days 1-4. She was treated with a second course of 5-AZA against MDS, but CD20 expression was not restored in the FL cells in her peripheral blood (Fig. 2).

## Discussion

The loss of CD20 expression is often observed after rituximab treatment in CD20-positive NHL [1, 2]. In the present case, the expression of CD20 protein gradually lost negative after bendamustine and rituximab treatments. Although a loss of CD20 expression is often associated with transformation in the FL to diffuse large B-cell lymphoma (DLBCL) [1, 2], the histology remained FL at the time of the loss of the CD20-negative transformation. This suggests that the phenomenon of CD20 expression does not necessarily indicate a transformation from FL to DLBCL.

Our patient’s FL was complicated by therapy-related MDS, and 5-AZA was administered for MDS. Following the first cycle of 5-AZA treatment, a small proportion of the CD20-negative cells exhibited CD20 expression. We also observed the risk of lymphoma cell stimulation due to 5-AZA. However, CD20 expression was not restored in the FL cells in her peripheral blood after the second course of 5-AZA. One possibility was that GDP-rituximab might have eliminated the FL clone, which restored the CD20 protein by 5-AZA. Another possibility was that the FL cells might have obtained a new chromosomal abnormality.

Recent studies demonstrated that CD20 protein and its messenger ribonucleic acid (mRNA) expression were enhanced in a CD20-negative transformed cell line RRBL1 in a culture with 5-AZA *in vitro* [1, 4]. 5-AZA upregulated CD20 due to the stimulation of transcription factors that stimulate MS4A1 by the regulation of the CpG methylation of gene promoters. This phenomenon restored the sensitivity of the FL cells to rituximab [1, 3, 4]. Our case further proved these *in vitro* findings *in vivo*. However, the effects of 5-AZA on CD20 induction were partial. *In vitro* studies demonstrated that the maximal effect of 5-AZA for the expression of the CD20 protein was observed 3 days after 5-AZA treatment [1, 4]. Therefore, a much earlier evaluation of CD20 expression after 5-AZA and earlier administration of rituximab following 5-AZA therapy may be required.

## Conclusions

Although there was partial upregulation of CD20 expression after 5-AZA treatment, it was not observed after a second administration of 5-AZA; we also observed lymphoma cell stimulation due to 5-AZA. Further studies must address these issues.

## Consent

Written informed consent was obtained from the patient for publication of this case report and any accompanying images. A copy of the written consent is available for review by the Editor-in-Chief of this journal.
